# Pediatric Multiple Sclerosis—A Challenging Demyelinating Disease: Case Report and Brief Review of the Literature

**DOI:** 10.1155/2012/684064

**Published:** 2012-07-11

**Authors:** Regina Célia Ajeje Pires de Albuquerque, Raquel Siqueira Leonel de Paula, Manuelina Mariana Capellari Macruz Brito, José Roberto Lopes Ferraz Filho, Lucas Crociati Meguins

**Affiliations:** ^1^Division of Neuropediatrics and Metabolic Diseases, Department of Pediatrics and Pediatric Surgery, Hospital de Base da Faculdade de Medicina de São José do Rio Preto (FAMERP), 5544 São José do Rio Preto, SP, Brazil; ^2^Department of Pediatrics and Pediatric Surgery, Hospital de Base da Faculdade de Medicina de São José do Rio Preto (FAMERP), 5544 São José do Rio Preto, SP, Brazil; ^3^Department of Neurological Sciences, Hospital de Base da Faculdade de Medicina de São José do Rio Preto (FAMERP), 5544 São José do Rio Preto, SP, Brazil; ^4^Department of Radiology, Hospital de Base da Faculdade de Medicina de São José do Rio Preto (FAMERP), 5544 São José do Rio Preto, SP, Brazil; ^5^Department of Neurological Sciences, Hospital de Base da Faculdade de Medicina de São José do Rio Preto (FAMERP), Rua Pedro Palotta, 101/31B, 15092-205 São José do Rio Preto, SP, Brazil

## Abstract

Multiple sclerosis (MS) is an inflammatory, demyelinating, neurodegenerative disorder of the central nervous system (CNS) of unknown etiology. The peak onset is between age 20 and 40 years and usually affects more women than men. Although much knowledge has been achieved on the diagnosis and treatment of adult patients with MS, it remains a matter of debate and controversy in childhood. We present a case of MS in 9-year-old girl, review the current state of the knowledge on pediatric MS, and discuss the available tools for the diagnosis and treatment.

## 1. Introduction

Multiple sclerosis (MS) is described as an inflammatory, demyelinating, neurodegenerative disorder of the central nervous system (CNS) and is uncommonly seen in pediatric patients [1–4]. Pediatric MS represents about 2.2% to 4.4% of all MS cases, in late childhood, affects more girls than boys, and is characterized by a relapsing-remitting course in almost cases [5–8]. Although, much knowledge has been achieved on the diagnosis and treatment of adult patients with MS, it remains a matter of debate and controversy in childhood.

The aim of the present report is to describe the case of 9-year-old girls with MS and discuss the current knowledge for the diagnosis of MS and the therapeutic possibilities.

## 2. Case Report

**Figure 1:**
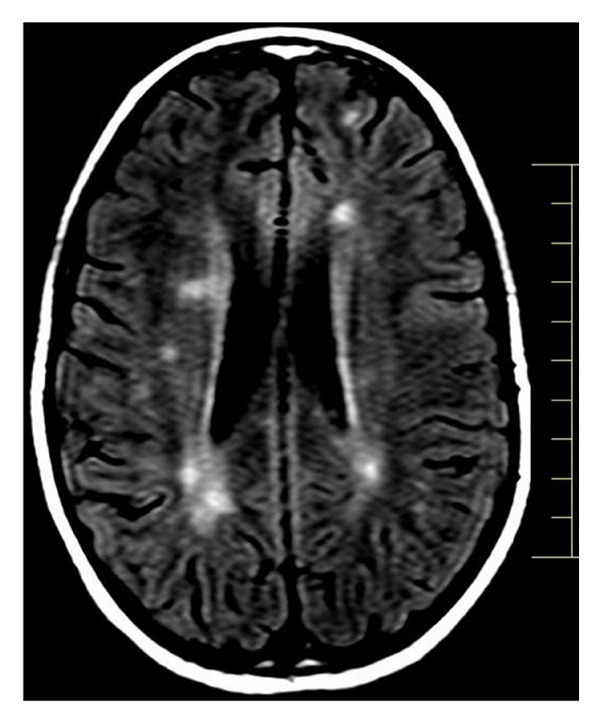
Brain MRI showing multiple periventricular lesions.

**Figure 2:**
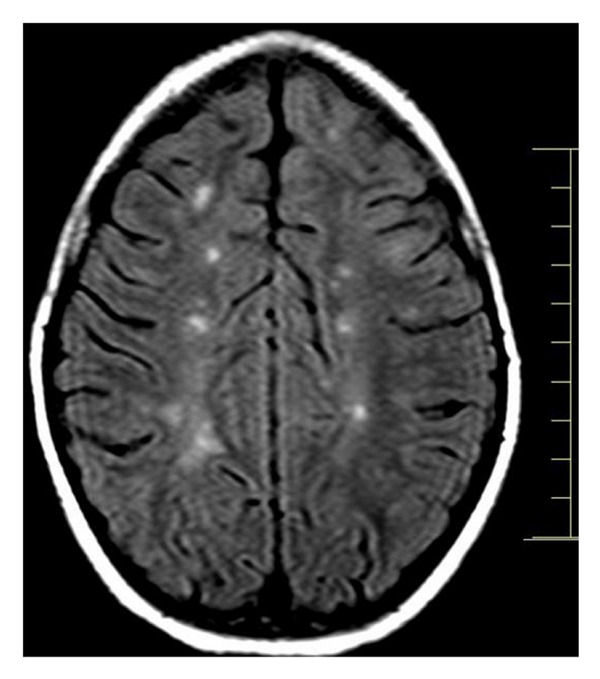
Brain MRI showing multiple juxtacortical lesions.

**Figure 3:**
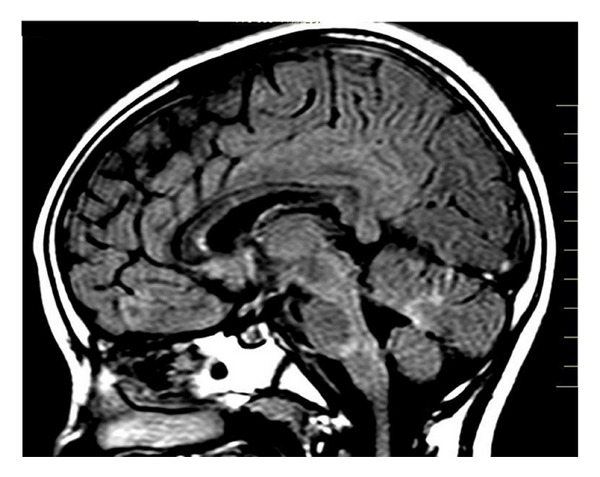
Brain MRI showing multiple infratentorial lesions.

**Figure 4:**
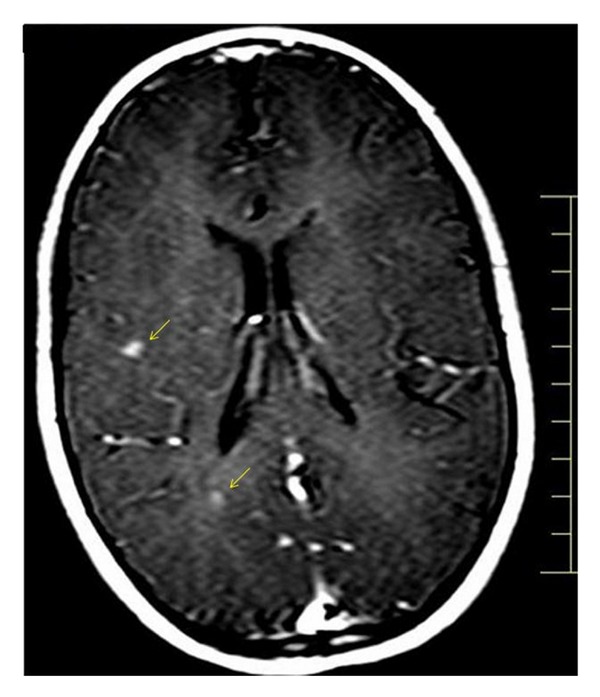
Brain MRI showing multiple gadolinium-enhancing lesions (arrows).

A 9-year-old girl was referred for neurological examination due to sudden onset of diplopia. The patient affirmed that after waking up on that morning, she started to feel visual impairment in which an object was seen as two while having both eyes open. She also said that the distance between the two objects enlarged when looking to the left. Her past medical and familial history was unremarkable for any degenerative or neurological disorder. General clinical assessment of the patient was found within normal limits. Neurological examination revealed convergent strabismus and no movement of the left lateral rectus muscle. The remainder of the exam was essentially normal, including visual fields and routine ophthalmoscopy. Blood laboratory exams and electrocardiogram were found to be normal. Magnetic resonance imaging (MRI) of the brain showed multiple lesions affecting the periventricular, juxtacortical and infratentorial regions (Figures 1, 2, and 3). Brain MRI also showed gadolinium-enhancing and nonenhancing lesions (Figure 4). A cerebrospinal fluid analysis was unremarkable. As no other structural abnormalities were identified, a diagnosis of multiple sclerosis (MS) was made and the neurological symptom was attributed to it. Based on this diagnosis, we started pulse corticosteroid therapy with methylprednisolone during three days. The girl did not recover after 6 months of the diplopia and is currently being followed on outpatient appointments.

## 3. Discussion

Multiple sclerosis (MS) is characterized as an inflammatory autoimmune disorder of the central nervous system (CNS) in which the fatty myelin sheaths around the axons of the brain and spinal cord are damaged, leading to demyelination and scarring [9–11]. It was first described by Jean-Martin Charcot, a French researcher, in 1868 [12], and affects more women than men [13]. Its incidence if found to be 5.68/100.000 per year and the pediatric population accounts for about 2.2% to 4.4% of all MS cases [5–8, 14]. Pediatric MS represents a challenging diagnosis to those that deal with pediatric care, mainly because it presents proper and different characteristics from adults.

Swanton and colleagues, in 2007 [15], and Montalban and colleagues, in 2010 [16], have made important contribution on the diagnosis of MS proposing simpler criteria allowing easer MRI evidence for dissemination in space (DIS) and dissemination in time (DIT) to be used in patients who present with clinically isolated syndrome (CIS), respectively. In May 2010, in Dublin, Ireland, on the third review of the McDonald criteria, the International Panel on the Diagnosis of Multiple Sclerosis (the Panel) not only accepted the simplified methods but also affirmed that the new criteria will also serve well for most pediatric MS patients, especially those with acute demyelination presenting as CIS [17].

 According to the Panel [17], DIS can be demonstrated with at least 1 T2 lesion in at least 2 of 4 locations considered characteristic of MS: juxtacortical, periventricular, infratentorial, and spinal cord. Additionally, DIT can also be confirmed in patients with typical CIS with a single MRI study that demonstrates DIS and both asymptomatic gadolinium-enhancing and nonenhancing lesions. In the present report, our patient suddenly complained of diplopia resulting from left VI cranial nerve palsy consistent with a clinical isolated syndrome affecting the brainstem. Brain and spinal cord MRI study revealed multiple lesions consistent with DIS and DIT additional criteria to MS.

Currently available first-line diseases-modifying therapies to MS for adults, including interferon *β* and glatiramer acetate, have not been approved by FDA for the treatment of children with MS [1, 18]. However, it has been proved to be safe and well tolerated in pediatric population [19–21]. Probably, different biology in terms of drug metabolism, immune mechanisms, and incomplete maturity of the central nervous system is responsible for the diverse response to treatment in children; therefore further investigation is required to assess the better clinical approach of pediatric patients. Our patient did not present a good clinical response to pulse corticosteroid therapy. She is currently being followed on outpatient appointments.

In conclusion, given the distinct features and substantial variability of symptoms in pediatric patients, a high clinical awareness to the possibility of MS diagnosis is necessary, seeking help from experts in central nervous system demyelinating diseases.
